# Causal criteria and counterfactuals; nothing more (or less) than scientific common sense

**DOI:** 10.1186/1742-7622-3-5

**Published:** 2006-05-26

**Authors:** Carl V Phillips, Karen J Goodman

**Affiliations:** 1 University of Alberta School of Public Health, Edmonton, Canada; 2 Department of Medicine, Faculty of Medicine and Dentistry, University of Alberta, Edmonton, Canada

## Abstract

Two persistent myths in epidemiology are that we can use a list of "causal criteria" to provide an algorithmic approach to inferring causation and that a modern "counterfactual model" can assist in the same endeavor. We argue that these are neither criteria nor a model, but that lists of causal *considerations *and formalizations of the counterfactual *definition *of causation are nevertheless useful tools for promoting scientific thinking. They set us on the path to the common sense of scientific inquiry, including testing hypotheses (really putting them to a test, not just calculating simplistic statistics), responding to the Duhem-Quine problem, and avoiding many common errors. Austin Bradford Hill's famous considerations are thus both over-interpreted by those who would use them as criteria and under-appreciated by those who dismiss them as flawed. Similarly, formalizations of counterfactuals are under-appreciated as lessons in basic scientific thinking. The need for lessons in scientific common sense is great in epidemiology, which is taught largely as an engineering discipline and practiced largely as technical tasks, making attention to core principles of scientific inquiry woefully rare.

## Background

An interesting persistent myth in epidemiology is that Austin Bradford Hill, the committee preparing the original United States Surgeon General's report on smoking, Mervyn Susser, or other authors have provided us a set of criteria for identifying cause-effect relations. This notion is remarkably robust given that these lists clearly do not meet usual definitions of criteria, which imply some sort of rule or test. Even when authors who invoke the "Bradford Hill criteria" yield to the scolding of various authors (including us [1]), and dutifully use Hill's word – "considerations" – rather than "criteria", they still seem to be in search of the elusive criteria.

A mythology to come into vogue more recently is that there is some "counterfactual model" that can help us to better recognize and understand causation in epidemiology. Just as causal criteria are not criteria, formal presentation of counterfactuals does not meet the definition of model, which can be thought of as a schematic or representation that captures part of the essence of a more complicated system in a way that leads to emergent properties.

In these pages, Höfler [2] took on the goal of trying to better understand Hill's considerations [3] by invoking a counterfactual model. As might be surmised from the above, we do not consider this to be a promising pursuit. We argue that causal considerations and the counterfactual conceptualization are both useful, but not in ways that support an analysis like Höfler's. Nevertheless, Höfler does provide what is possibly the best one-sentence assessment of the concept of causal criteria, and nicely (though perhaps inadvertently) helps argue the case that causal considerations and counterfactuals are, more than anything else, guideposts on the road to common sense.

## Analysis

### Not criteria

"Criteria" is sometimes defined overly broadly to include anything you might want to think about when making a decision (that is, as a synonym for "considerations"). But most definitions include a reference to a test, basis for judgment, or condition (and anyone trying to "apply" a set of criteria to make a determination must have such a definition in mind). Clearly, causal considerations do not meet these tighter definitions of criteria. There is no method for determining whether or how well each consideration is met (for example, researchers seem able to concoct some biological story to explain any association in their data; how absurd does it have to be before there is no biological plausibility?), let alone how we would aggregate any such scores for individual considerations into an ultimate decision about cause and effect. This tends to be obscured when commentators' main criticisms are that the proposed conditions are neither necessary nor sufficient, overlooking the salient fact that they are not actually well-defined conditions (and thus can be neither necessary nor sufficient, nor can they be non-necessary or non-sufficient).

With that in mind, it is instructive to consider the implications of authors providing worldly examples of causal conditions being met as evidence for the conditions being either informative or misleading, or of attempts like Höfler's to improve the application of criteria. Those authors clearly have in mind some standard for judging whether a condition is met and whether a relationship is causal. The latter assessment must be independent of the criteria (since it is meant to validate the usefulness of the criteria) and, most importantly, is presumably meant to be something most readers would agree upon. This suggests a presumption of shared common sense. Poole [4,5], inspired by Thomas Kuhn [6,7], suggests that rather than criteria, causal considerations are "values" which different scientists can hold to different degrees. Values are bases for making worldly conclusions, but tend to lack scoring systems and other elements of algorithms, and any claims based on them are subject to interpretation and scrutiny. Indeed, the empirical and experimental evidence Poole cites makes clear that epidemiologists' interpretations of the considerations vary substantially [8-14]. But debates among scientists about which values are legitimate suggest a feeling that there should be some shared scientific common sense, rather than persistent heterogeneity of values.

Neither Hill's nor any other list can codify common sense, but it can introduce some of it and thereby provide a starting point. This is quite useful since common sense is disturbingly uncommon and thus in need of whatever help it can get. For researchers who fail to consider, say, consistency across studies or coherence with previous knowledge in their assessment of causation, and proudly declare that "our research is the first to show that exposure E causes disease D, contrary to numerous previous findings," Hill's lesson in common sense has immediate value. Attending to Hill's or others' causal considerations would encourage anyone writing, "our research is the first to show X...," to follow it – as they almost always should – with, "...so X is probably not true."

Of course, common sense is most useful in simple cases, while modeling (e.g., drawing diagrams of causal pathways) becomes more critical as a system becomes more complex. Höfler observes that "the heuristic value of Hill's considerations converges to zero as the complexity of a causal system and the uncertainty about the true causal system increase" [2]. This may be the definitive observation about causal criteria/considerations. To venture a simpler paraphrase, lists of causal considerations are pretty good rules of thumb when the system being assessed is simple, but in cases where an assessment of causation demands more than common sense, these lists are not going to be terribly useful. Höfler goes on to try to improve on Hill's list to make it more useful in complicated cases, but we think he was right the first time: in a complicated system the list can only serve as a tool for teaching scientific common sense, and no matter how we try to dress it up, it cannot serve as a checklist, algorithm, or method.

### Not a model

The use of the term "model" in the previous paragraph illustrates its meaning. Causal diagrams take as inputs some of the known or postulated elements of a worldly system of causes and effects and schematize them in a way that new knowledge (i.e., beyond the inputs themselves) can be extracted. In this sense, a small three-dimensional scale version of an airplane is a model (because, for example, we can put it in a wind tunnel and learn something about the actual airplane that we did not know when we made the model) but a photograph of the plane is not a model (at least not in any obvious way). Neither is the phrase "heavier-than-air, fixed-wing, self-propelled flying vehicles" a model. The phrase is informative about airplanes, but in a different way from a model: it is the definition of airplanes. We need to have that phrase (or some variation) in mind before it even makes sense to talk about airplanes, let alone model them. It might be useful to refer back to the definition if, during an assessment of airplanes, we somehow lost touch with the class of things we are talking about. But the definition is not a model; it does not offer a way to extract any information that is not merely an input into it, such as assessing how airworthy a particular airplane is. Indeed, it cannot in itself help us determine if a particular object really makes the cut (e.g., that it can really fly).

In that spirit, what many authors, including Höfler, mistakenly call the "counterfactual model of causation" can easily be seen to be a definition, not a model. There is an extensive philosophical literature on what the verb "cause" means (including when it is implicit in many other verbs or phrases such as "increases", "leads to", and "protects against" [15]). These discussions include alternative definitions as well as arguments that the word actually has no definitional teeth. But in the everyday practical world of epidemiology (a field we define broadly to include empirical and experimental research on diseases and health-related exposures with people as the unit of analysis), we would venture to say that most everyone who uses causal language is implicitly invoking the counterfactual definition, "but for E, D will not occur or would not have occurred, but given E it will/would have" (described in more detail and with more symbolic logic by Höfler and many other authors; see in particular Maldonado and Greenland's "Estimating Causal Effects"[16]). We cannot think of any use of the word "cause" in epidemiology (in the research and its policy implications, excluding purely philosophical discussions) where the author seemed to have something else in mind.

This does not mean that careful attention to the definition is worthless. Maldonado, a leading proponent and teacher in epidemiology of the formal counterfactual definition and its implications (and who refers to the "counterfactual approach", "concept", or "definition", but not "model"), has pointed out that it aids us in, among other things, specifying epidemiologic questions, assessing which statistics are genuine measures of effect, designing studies, and defining confounding. Much of this, however, is arguably scientific common sense (see further discussion below), not of the "values" sort, but in the form of first- or second-order logical inferences that scientists should intuitively grasp. But, again, since common sense may be woefully uncommon, the formalizations by Maldonado and others are valuable.

### Invoking counterfactuals in pursuit of better causal criteria

We thus agree with Höfler's assessment that Hill probably had a counterfactual concept – *definition*, not model – of causation in mind (consciously or subconsciously) when he gave his famous (and under-appreciated [1]) talk [3], not merely because of some specific phrase he used but because it is difficult to imagine anything else he could have had in mind. Though Höfler argues that "counterfactual causality [presumably meaning the counterfactual definition of causality] ... only became standard in epidemiology from the 1980s" [2], it seems very unlikely that epidemiologists (or economists or statisticians, for those who prefer those characterizations of Hill) had some other definition in mind before that. Like Newton "discovering" gravity, those who formalized the definition of causation in philosophy, mathematical statistics, and applied sciences did so in a context in which most people already grasped the basic idea and made use of it (to make scientific inferences or to keep from floating away into space).

With the counterfactual concept providing merely the definition, one that Hill shared with most of us, it seems unlikely that it can teach us much new about Hill's list. Indeed, it does not appear that Höfler finds any teeth in the notion of counterfactuals.

Höfler's analysis begins with the strength of association condition, a particularly good heuristic when a system is simple (e.g., a large, well-designed randomized trial with results that are easily measured soon after the intervention). But strength of association is considerably less definitive when confounding and other errors add complexity to our assessment. Höfler addresses the uncertainty that results from study errors, asking "Would the interval estimate that properly accounts for not only random, but also systematic error...allow for the desired conclusion...?" adding, "high uncertainty about bias parameters requires larger associations than modest uncertainty does." That is, whether or not an association is strong is a matter of context.

There are analytic methods being developed to put some numbers to that context, and we appreciate and encourage the attention to quantification of epidemiologic uncertainty from errors other than random sampling, a line of thinking in epidemiology that one of us helped to launch [17] (see endnote 1). But despite the fact that this line of thinking sprang from Maldonado's work on causal contrasts (a line of thinking he traces proximately to Greenland and Robins [19], and which also traces to Rubin, Neyman, Hume, and other thinkers) we have to say that Höfler's assessment seems to have nothing to do with counterfactuals. It primarily supports his thesis that complicated systems defy the simple rules of thumb. This conforms to what we have argued previously: uncertainty about input assumptions (e.g., the assumptions that measurement is accurate and confounding is controlled for) is almost always ignored in epidemiologic results, and people (including experts) have been shown to be quite bad at quantifying the possible magnitude of error without mathematical aids [17,18,20,21]. Höfler tries to improve upon the simplest statement of the strength of association consideration, but provides nothing that is any more operationalizable, leaving us again with values or common sense.

Höfler structures his analysis around "what if" questions, calling them counterfactuals, but this gets no apparent traction from formally representing the counterfactual definition or pursuing its implications. For example, after observing that the consistency criterion suffers because different studies of different populations are expected to produce inconsistent results, Höfler asks questions including, "If the causal effect varied across the studies," (presumably actually meaning if it varied across the different study populations, exposure definitions, etc. that are implicitly defined by the studies) "would one expect to observe different associations...?" This is a useful lesson on consistency, replacing the hobgoblin of foolish consistency with systematic prediction of inconsistency. This is used when, for example, authors find it reassuring that the association with an exposure is stronger for histologically-confirmed cancers than it is for an alternative (presumably noisier) definition of disease status. Since we would expect to see a stronger association (more likely than not) when there is less (independent, non-differential) measurement error, this inconsistency could make us more comfortable with a causal conclusion. However, the role of counterfactuals in this lesson, beyond the implicit definition of causation, is unclear.

It appears that any value in Höfler's analysis lies not in counterfactuals, but in *hypotheticals *– that is, *ex ante *hypotheses about what data would show if a certain assumption were true. Perhaps this puts a finer point on "common sense," replacing it with the systematic scientific thinking that epidemiology needs much more than it needs improved causal checklists. Our example, that different disease definitions should result in inconsistent associations (in a predictable way), introduces a testable hypothesis. Höfler presents another under the specificity criterion, borrowing the example [22] that wearing helmets, if it reduces injury rather than just being a proxy for an unmeasurable tendency to act more carefully, should result in reduced injuries of the head, but not other body parts. Both of these examples are useful and, though immediately compelling when presented, may be a step beyond mere common sense. There is clearly value in teaching health researchers to think more about proposing and testing hypotheses (in the genuine sense discussed below). Conversations about evidential clues (e.g., lists of causal considerations) provide one good starting point for teaching such lessons. Indeed, there is every reason to believe that this was what Hill was trying to do when he gave his talk.

Problems result when people mistakenly treat Hill's lessons as being from the wrong branch of philosophy, interpreting them as rules of logical rather than worldly philosophy of science and the ethics of decision making [1]. Höfler (quoting Rothman and Greenland [[23], p.27]) notes that one condition – that cause must precede effect – is "the only *sine qua non *for a counterfactual effect" (see endnote 2). Although temporal ordering is a necessary condition according to the physics we understand, or even simple semantics (the condition follows directly from some phrasings of the definition of cause), this does not make this consideration any more or less useful than others as a lesson in common sense. Lessons such as, "if a measured upward trend in cancer rates leads (rather than lags) the measured increase in the exposure that you think is causing it, you are probably wrong about your causal conclusion," are not fundamentally different from other common sense applications of Hill's considerations.

### The need for lessons in common sense

Why do health researchers, seemingly much more than those in other fields, cling to rules for assessing causation to the point that we have several such lists as well as a secondary literature that tries to assess and improve the rules? Why, as suggested by Kaufman and Poole [5], did Susser [24] provide five strategies for assessing causation – strategies for testing hypotheses alongside his list of causal criteria – but respond to greater interest in the criteria list by subsequently focusing on the list and de-emphasizing the other strategies? Part of the answer may lie in the emphasis on observational data (since well-designed interventions provide simpler support for causal claims, at least for some types of inquiry). However, this cannot be the whole story, since physics and biology (to say nothing of economics) quite often rely on observation alone.

Probably more importantly, the desire to find answers to countless different policy, social science, and biological questions creates the desire to study something once (in a particular population, at a particular time, with particular variable definitions), declare an answer, and move on. This does not provide much opportunity to actually test hypotheses. It encourages health researchers to conduct simplistic statistical calculations that are described in the language of hypothesis testing, and mistake this for actually testing a worldly hypothesis. It discourages genuine hypothesis testing, along the lines of, "If we have observed a true causal relationship, then we would also expect to see.... Let's do more research to check that before reporting our result." We would certainly expect such testing from another science before it declared, say, the discovery of cold fusion or that unfettered free markets make people's lives better (bad examples, perhaps – call them exceptions that emphasize the value of the rule).

Epidemiology sees few studies designed to chip away the ambiguities resulting from the Duhem-Quine problem (which, roughly speaking, is the quandary that any study used to test a particular claim is simultaneously testing many ancillary hypotheses about the study methodology – e.g., that the right measures were used, the instruments do what they are supposed to – and thus we cannot be sure the observed result informs the causal hypothesis of interest). Studies are seldom repeated with improved (or even different) instruments (see endnote 3). Validation studies are occasionally conducted, quite often finding substantial measurement error, but the results are almost never incorporated into the primary analysis. Even easy analyses that require no further fieldwork, such as assessing whether an effect estimate is highly dependent on the particular functional form used in the quantitative analysis (i.e., statistical model assumptions, cutpoints for categorizing variables, etc.) are rarely reported.

Similarly, new studies on a topic almost never actually replicate a result, failing to take the simple step of using a previously defined model on a different dataset. Instead they use a new *ad hoc *model, ensuring that too many things vary at once for us to be able to distinguish our result of interest from the ancillary hypotheses. (Epidemiologists may find this point most familiar in the context of meta-analysis, where careful researchers often discover that there are many more dimensions of variation among study methods than there are studies.) Claims of causation in this context are rather strained, whatever models, criteria, or equations we might have.

What is worse is that there is not just negligence about doing good science, but actual attempts to subvert it. Not only is there no attempt to conduct and report alternate analyses that test the robustness of a statistical model and use the findings of such tests to address uncertainty, but in many cases, many statistical calculations are performed and the one reported is chosen *because *it is an outlier (i.e., because it shows a dramatic result), making it most likely to be an artifact of false ancillary hypotheses about the model [25]. Thus, not only do researchers fail to further test the causal conclusions they draw based on their data, but their causal conclusions are often not even supported by their data (since most calculations using the data would produce less extreme results than the ones reported). This approach violates common-sense norms of scientific inquiry, including Hill's often-overlooked preamble consideration, that the data must show an association in the first place. Unfortunately, this subversion is not terribly surprising when the desire to get an interesting result is not tempered by concern about replicablility and consistency (there is very little chance anyone will ever attempt to actually replicate a result, and health researchers show an unfortunate tendency to cite an outlier result as evidence of an association, regardless of how many other studies found a null association), or by real scientific training that imparts an ethic about what constitutes good science.

The desire to substitute what is ostensibly a checklist of criteria for real scientific analysis and thinking seems to reflect the practice of health science rather than the nature of epidemiologic data. Just as most health science ethics classes offer legalistic checklists, rather than serious analysis of ethics, most epidemiology pedagogy offers a set of tools, without much scientific thinking. There is nothing inherently wrong with training people to be engineers – skilled users of complicated tools they can adapt to specific practical applications. The field of epidemiology was largely created by members of one field of engineering, physicians (who, incidentally, constituted most of Hill's original audience, a telling bit of context that is usually ignored), with sage advice from various sciences (Hill's approach reflects his background as an economist).

Epidemiologic training is almost always designed to create engineers, practitioners who produce tangible results, but who devote little attention to questions about the nature of inquiry or scientific truth. Moreover, health science practice is dominated by those who lack even adequate skills in epidemiologic engineering; they tend toward rote application of particular techniques and use of off-the-shelf software they do not really understand – a pattern that describes technicians, not engineers or scientists. One might be tempted to counter that most practitioners of every science spend most of their time carrying out technical tasks. But the education and expectations of scientists in most fields include fully understanding the models and methods they use and trying to advance the methods in pursuit of inquiry; those who mechanically operate conceptual or physical tools they cannot explain and would not have been able to create from scratch are not generally called "doctor" and do not dominate the scientific output of other fields. This is particularly true in sciences that are as immature as modern health research (see endnote 4).

In this context, health "science" tends to avoid and even disdain scientific thinking: There is little interest in rigorously challenging conclusions before expressing comfort with them. Initiating vigorous learned debate or suggesting that researchers should be required to defend their claims against criticism is frequently considered impolite or even hostile. Pursuit of better methods of research and analysis, despite how terribly primitive our methods are, is considered an esoteric sideline rather than the lifeblood of the science. Results of published studies are cited as if they were definitive, without adequate regard to the quality of the research, even when there is clear reason for doubt. Methods sections in research reports do not provide even remotely sufficient detail to understand what was done. Datasets are seldom re-analyzed, no matter how important the implications. And on top of these problems (or perhaps because of them), the cursory peer-review process is treated as if it – rather than a crucible of further study and debate – determines the truth of a claim.

Epidemiology education is seldom designed to produce scientists. In our experience, if two professors present conflicting views on proper methodology, students typically react with discomfort, or even hostility, insisting that someone just tell them what is right so they can use it and move on. From what we have seen, most training in epidemiology indulges (or even helps create) this mindset, catering to students who are clearly budding technicians, not scientists. Students are usually taught to use computational black-boxes and describe the results with rote language. Some of them want to be scientists, and try to engage in scientific analysis and inquiry, but being taught (or even forced) to conform to the dominant modes of practice makes that difficult. A student who masters a typical program in epidemiology will be a competent engineer, but will have learned little about the nature of scientific inquiry.

To be sure, engineers could be considered the bedrock of modernity and technicians undoubtedly produce more total day-to-day benefits than scientists, so this is not a pronouncement about comparative worth. But it does explain why scientific thinking needs a boost in the field. We would be surprised if even 1/1000th of the person-time spent doing epidemiology is devoted to critical analysis.

## Conclusion

It is in this context, a field of scientific inquiry that is dominated by non-scientists, that lessons in scientific common sense have immense value. Four years before "Estimating Causal Effects" was published, Maldonado presented a seminar on the usefulness of formalizing counterfactuals, and following it one of us (CVP), new to epidemiology at the time, asked, "what part of that was I not supposed to already know?" In retrospect, the question clearly missed the point: Like Hill's contemplations, the formalization of counterfactuals is not a new discovery or even a new lesson, but rather an articulation of a concept that deserves more attention (or basic awareness) than it gets in health research. Indeed, we emphasized the need for further analysis of what is "known" in the field (in the sense of having been said some time, in some way), but seem to be remembered far too infrequently, as a major reason for starting a new journal [26,27]. As every teacher knows, spending time contemplating previous lessons is usually a lot more valuable than introducing a novel idea every minute of every lecture.

Attention to a formal definition of causation and to a list of clues that might help us draw conclusions about causation can be valuable. Such attention can help promote the active thinking that leads to scientific common sense. So long as the message is interpreted as the need to contemplate and investigate before drawing scientific conclusions, these lessons are valuable.  But when they degenerate into black-box algorithms, this enables health researchers to avoid the intellectual work of being scientists.

### Endnote 1

We find it unfortunate that Höfler used the term "Monte Carlo sensitivity analysis," to describe some uncertainty quantification methods. Phillips has pointed out that this is a misnomer since those methods differ fundamentally from sensitivity analysis and "Monte Carlo" confuses the calculation tool with the analysis [18].

### Endnote 2

It is worth mentioning that the temporality condition is also a perfect fit for counterfactual-avoiding definitions of causation such as "predictable patterns of one event following another", suggesting again that nothing is learned about causal considerations by invoking counterfactuals.

### Endnote 3

Ironically, as we were writing this paper, one of us attended a workshop on getting health research grants from the Canadian government; part of the advice was that the exposure-disease relationship being studied needs to be novel. The message was that checking the robustness of previous results was such a low priority that it would not attract this funding.

### Endnote 4

To add concreteness to the point about conceptual machinery, consider how many of those who are considered scientists in epidemiology ever learned how to calculate the statistics they report without depending on a black-box software package or, for that matter, how many can even define confounding, let alone explain why their mathematical model was the best choice or calculate the impact of measurement error. In a science that is profoundly still under development, we would expect that scientists would be educated and conversant in the entire process of inquiry so that they could contribute to the development. Epidemiology is clearly immature and under development: most epidemiologic research in history has been done within the lifetimes, often even the professional lifetimes, of current researchers, and the list of known glaring failures of the methods is long.

## Competing interests

The authors have previously written on related topics and have an incentive to support their previously published points of view. Their views are influenced by a high level of frustration with current scientific standards in health research. CVP was trained largely as an economist, and his praise for Hill somewhat reflects a shared disciplinary outlook.
